# Molecular characterization, developmental expression, and modulation of occludin by early intervention with *Clostridium butyricum* in Muscovy ducks

**DOI:** 10.1016/j.psj.2021.101271

**Published:** 2021-05-21

**Authors:** Wentao Lyu, Hua Yang, Na Li, Lizhi Lu, Caimei Yang, Peihua Jin, Yingping Xiao

**Affiliations:** ⁎ State Key Laboratory for Managing Biotic and Chemical Threats to the Quality and Safety of Agro-products, Institute of Agro-product Safety and Nutrition, Zhejiang Academy of Agricultural Sciences, Hangzhou 310021, China; † College of Animal Sciences & Technology, Zhejiang A & F University, Hangzhou 311300, China; ‡ Institute of Animal Husbandry and Veterinary Science, Zhejiang Academy of Agricultural Sciences, Hangzhou, 310021, China

**Keywords:** Muscovy duck, occludin, *Clostridium butyricum*, gene expression

## Abstract

Occludin is an important component of tight junction proteins and has been extensively studied in animals such as mice, chickens, geese, and pigs. As one of the most important waterfowl species in China, Muscovy duck (*Cairina moschata*) is an important economic animal for meat. However, research on the occludin gene in Muscovy duck is lacking. In the present study, Muscovy duck occludin cDNA was cloned for the first time. The length of the cDNA was 1,699 bp, and it showed a high sequence similarity with the *Anser cygnoides domesticus* and *Gallus gallus* occludin genes. The occludin gene was differentially expressed in the tissues of healthy ducks. The highest and lowest expressions of occludin were observed in the crop and the spleen, respectively. After the oral administration of *Clostridium butyricum* (**CB**), the occludin expression in the ileum of 7-day-old Muscovy ducks was significantly upregulated and subsequently showed a decreasing trend in 14-day-old Muscovy ducks. Under the early intervention of CB, no significant difference was observed in the occludin expression of cecum between the control and CB group. Collectively, these results suggest that CB plays an important role in regulating the expression of the occludin gene in Muscovy ducks, and adding CB in feed may maintain the intestinal barrier of ducks by regulating the expression of occludin.

## INTRODUCTION

As an important economic animal species, Muscovy ducks (*Cairna moschata*) are popular among consumers for its unique flavor, and the demand for duck meat is increasing year by year. However, Muscovy duck industry is currently threatened by a variety of intestinal diseases, which cause huge economic losses (Fujimoto et al., 2016; Wu et al., 2019; Shi et al., 2020). Therefore, ensuring the intestinal barrier of Muscovy ducks is of great significance to the stable development of the Muscovy duck industry.

The gastrointestinal (**GI**) tract is a series of hollow organs that form an important mucosal barrier for the host, which plays an important role in immune system and energy homeostasis (Bischoff et al., 2007; Okumura et al., 2018; Ying et al., 2020). As a complex structure, the intestinal barrier has many physiological functions, such as serving as a physical barrier, participating in nutrient digestion and absorption, and regulating immune response (Walker et al., 2014; Ying et al., 2020). The intestinal barrier has been demonstrated to protect the stability of the intestinal microenvironment from the invasion of exogenous pathogenic microorganisms and harmful factors (Cui et al., 2019). Tight junctions are components of the intestinal barrier and important molecular structures that maintain the barrier between intestinal epithelial cells and endothelial cells. This would maintain the intestinal barrier function and regulate intestinal permeability to provide a stable intestinal microecological environment for the host (Zeiller et al., 2009; Chen et al., 2017; Fang et al., 2018; Wu et al., 2019). The mucosal barrier is primarily composed of epithelial cells and many intercellular connections. Tight junction proteins include occludin, claudin, and Zonula Occludens (**ZO**), among which occludin is the most important part of tight junctions.

Probiotics are proved to enhance the intestinal mucosal barrier function (Resta-Lenert et al., 2006; Johnson-Henry et al., 2008; Gong et al., 2019; Gong et al., 2020). As one of the most popular probiotics in the market, *Clostridium butyricum* (**CB**) is a butyrate-producing bacterium that can repair damaged intestinal mucosa through the production of butyric acid (Duncan et al., 2002) and protect the intestinal epithelium by upregulating the expression of occludin, ZO-1 and Claudin-1 (Li et al., 2018). CB has been widely supplemented in poultry feeds including ducks. The effects of CB on poultry mainly include the growth performance, immune function, and gastrointestinal microflora in turn to the profile of short chain fatty acids in the cecum and to prevent poultry from infections (Liao et al., 2015; Huang et al., 2019; Zhan et al., 2019). Dietary supplementation of CB could improve growth performance, lipid metablism, and meat quality of Peking ducks and Cherry Valley ducks (Chen et al., 2018; Liu et al., 2018).

Currently, the occludin protein sequence has been reported in different animal species, including mice, pigs, chickens, and geese (Lu et al., 2015; Luo et al., 2017; Lucke et al., 2018; Yan et al., 2018; Alfajaro et al., 2019; Woo et al., 2019). However, there are limited reports on the sequence, function, and dynamic expression patterns of occludin in ducks. In the present study, for the first time, we reported the full-length cDNA and bioinformatic analysis for the occludin gene of Muscovy ducks. In addition, we examined the occludin gene expression patterns in different tissues and the temporal expression of occludin in the GI tract of Muscovy ducks. Moreover, the effect of the early inoculation of Muscovy duck with *Clostridium butyricum* on intestinal occludin gene expression was investigated.

## MATERIALS AND METHODS

### Animals and Tissue Sampling

Experiment 1: Eighty day-of-hatch Muscovy ducks were obtained from a commercial hatchery (Lanxi Hewang Breeding Co. Ltd., Lanxi, China). The ducks were raised in cages (10 ducks per cage in 0.52 m × 0.62 m × 0.52 m) and had ad libitum access to commercial diets and water as previously described (Lyu et al., 2021). Intestinal tissues were collected at d 1, 3, 7, 10, 14, 28, 49, and 70. Eight ducks were randomly selected (1 per cage) for each sampling date, and the tissue segments collected at the 14-day-old included brain, breast muscle, abdominal fat, liver, skin, heart, kidney, spleen, thymus, bursa of Fabricius, trachea, lungs, tongue, esophagus, crop, proventriculus, gizzard, duodenum, jejunum, ileum, cecum, and colon. The collected samples were immediately frozen in liquid nitrogen and then transferred to a -80°C freezer until RNA extraction.

Experiment 2: One hundred and sixty day-of-hatch Muscovy ducks were obtained from a commercial hatchery. Ducks were randomly divided into 2 groups. Each group had 8 replications and 10 ducks per replicate. All ducks were fed with a commercial starter diet (Lyu et al., 2021) in cages (10 ducks per cage). The experiment included 2 treatments, where ducks in the CB group were orally administered with 1 mL of a *Clostridium butyricum* suspension (2 × 10^9^ CFU/mL) after hatch (day 0) while ducks in control group received the same amount of saline at the same time (day 0). Administration was performed once a day and lasted for 3 d. *Clostridium butyricum* was provided by Miyarisan Pharmaceutical Co., Ltd (Tokyo, Japan). Eight ducks per group (1 per replicate) were randomly selected at d 7 and 14 for sample collection. Ileal and cecal segments were collected and preserved using the same method described above.

All of the experiments were conducted under the ethical guidance of animal care and use in the laboratory of the Zhejiang Academy of Agricultural Sciences.

### RNA Extraction and Reverse Transcription

Total RNA was extracted from different tissues with a TRIzol Plus RNA Purification kit (Thermo Fisher), and Superscript III First-Strand Synthesis SuperMix for quantitative RT-PCR (**qRT-PCR**) (Thermo Fisher) was used to synthesize the First Strand cDNA from total RNA following the manufacturer's instructions. The following reaction conditions were used: 25°C for 10 min, 50°C for 30 min, and 85°C for 5 min.

### Real Time Polymerase Chain Reaction (RT-PCR)

Primer Premier 6.0 was used to design qRT-PCR primers, which were subsequently synthesized by Bioengineering Co., Ltd. (Shanghai, China) (Table 1). RT-PCR was performed using PowerUp SYBRTM Green Master Mix (ABI). A SuperScriptIII First-Strand Synthesis SuperMix for qRT-PCR (Thermo Fisher) was used to perform the first-strand cDNA synthesis according to the manufacturer's instructions. GAPDH was used as an internal reference gene and the occludin cDNAs fragment was amplified from Muscovy duck RNA through a round of PCR. Each reaction was performed using the following thermocycling program: Predenaturation at 95°C for 1 min followed by 40 cycles of denaturation at 95°C for 10 s and annealing at 60°C for 25 s. Eight biological replicates were performed for each group, with triplicates for each sample. The relative expression levels of different target genes were calculated using the 2^−∆∆Ct^ method.

**Table 1:** Information of primers.

Primers	Sequences (5’-3’)	Product Size(bp)	Purpose
GAPDH-F	GGAGCTGCCCAGAACATTATC	141	Real-time PCR
GAPDH-R	GCAGGTCAGGTCCACGACA		
Occludin-F	CAGGTGTACAGCAGCAGCACTT	126	Real-time PCR
Occludin-R	GAAGCAGATGAGGCAGAGCAAGA		
rOccludin-F1	CAGCAAGGAGCTCGACAGCATCTCT	263	3’-RACE
rOccludin-F2	CAGTACCAGGACGTGGCAGAGGAATAC		
rOccludin-R1	GCCGTAGTAGCCGGAGCCGTAGTAG	293	5’-RACE
rOccludin-R2	GCAGGCGAAGATGGCGATGCA		
mOccludin-F	GGCGTGGTGAGGATCCTG	1278	Coding region amplification
mOccludin-R	CTTCTTGCTCTGGTAGTCG		

### 5’ and 3’ Rapid Amplification of the cDNA Ends (5’ and 3’RACE)

The RACE experiment was performed as previously described with minor modifications (Xiao et al., 2019). In detail, the first strand cDNAs for 5’ and 3’ RACE were synthesized using the GeneRacer kit (Invitrogen) and used as the template to amplify the 5’ and 3’ region cDNA fragment of occludin. PCR was performed using the gene-specific primers (Table 1) and the RACE primers contained in the RACE Kit. For the 5’ and 3’ RACE of occludin, the following program was used: 94°C for 2 min followed by 30 cycles of 94°C for 30 s and 66°C for 30 s.

### Comparison and Evolutionary Analyses of the Muscovy Ducks (Cairna moschata) Occludin

The occludin amino acid sequence of Muscovy ducks was compared with those from different representative animal species. The alignment was generated with the Multiple Sequence Alignment function in CLC Genomics Workbench 12. The height of the pink bar under the residues represents the conservation of the corresponding residue.

### Protein 3D Structure Prediction

The homologous structure modeling I-TASSER server (http://zhanglab.ccmb.med.umich.edu/I-TASSER/) was used to predict the 3D structure of the Muscovy duck occludin protein based on homology structure modeling as previously described (Yu et al., 2018). Briefly, structural similarity of 2 protein models was measured by the TM-score and RMSD from the I-TASSER server. Pymol program (http://pymol.sourceforge.net) was used to superimpose similarities between the predicted structure and template sequence. Global and per-residue model quality, assessed by C-scoring, provided a confidence score for estimating the quality of the predicted model by determining the significance of threading template alignments and convergence parameters of structural assembly simulations in I-TASSER.

### Phylogenetic Analysis

The phylogenetic tree of Muscovy duck occludin and potentially related genes was constructed using MEGA (version 10.1) with the Jones-Thorton-Taylor (**JTT**) model based on the neighbor-joining method with 1000 bootstrap replicates (scale bar is 0.10). The occludin protein sequence from Muscovy duck was compared with those from other representative species, which included number of amino acids and the percent identity (Table 2).

**Table 2:** Comparison of Muscovy duck (*Cairna moschata*) occludin and the occludin proteins from other species.

Species	NCBI reference sequence	No. of residues	Identity (%)
*Cairna moschata*	MT_420729	508	100
*Anser cygnoides domesticus*	XP_013055123.1	440	89.126
*Gallus gallus*	NP_990459.1	504	79.961
*Mus musculus*	NP_001347465.1	521	46.78
*Homo sapiens*	NP_001192183.1	522	48.728
*Capra hircus*	XP_017921166.1	522	46.125
*Sus scrofa*	NP_001157119.1	522	45.383
*Bos taurus*	NP_001075902.1	522	46.729
*Felis catus*	XP_019690751.1	521	47.547
*Equus caballus*	XP_023474005.1	520	45.351
*Danio rerio*	NP_997997.2	491	41.317

### Determination of Short Chain Fatty Acids (SCFAs) Content in Cecum by Gas Chromatography

SCFAs, including acetic acid, propionic acid, butyric acid, isobutyric acid, valeric acid and isovaleric acid, were measured in cecal content samples from each group at each time point by using gas chromatorgraphy as previously described (Gong et al., 2019). Briefly, 100 mg of cecal content was homogenized with 1 mL of sterile PBS. After being centrifuged for 10 min at 12,000 rpm and 4°C, a 500 μL of the supernatant was diluted with 100 μL of 25% (w/v) metaphosphoric acid solution. The mixture was incubated for 24 h at -20°C. Then the mixture was centrifuged for 10 min at 12,000 rpm and 4°C followed by the supernatant was collected and filtered through a 0.22 mm syringe filter. The prepared samples were injected into a Shimadzu GC-2010 ATF instrument with N_2_ as the carrier (pressure, 12.5 Mpa and flow, 18 mL per min), the temperature of the injector and detector was 180°C, and the column was gradually heated from 80 to 170°C at a rate of 4°C/min.

### Statistical Analysis

The data were analyzed by unpaired two-tailed Student's *t* test using SPSS 19. Differences where *P* < 0.05 were considered significant, while differences where *P* < 0.01 were considered extremely significant. All data were processed using GraphPad Prism 6.0.

## RESULTS

### Molecular Cloning of Occludin cDNAs from the Muscovy Ducks

**Figure 1:**
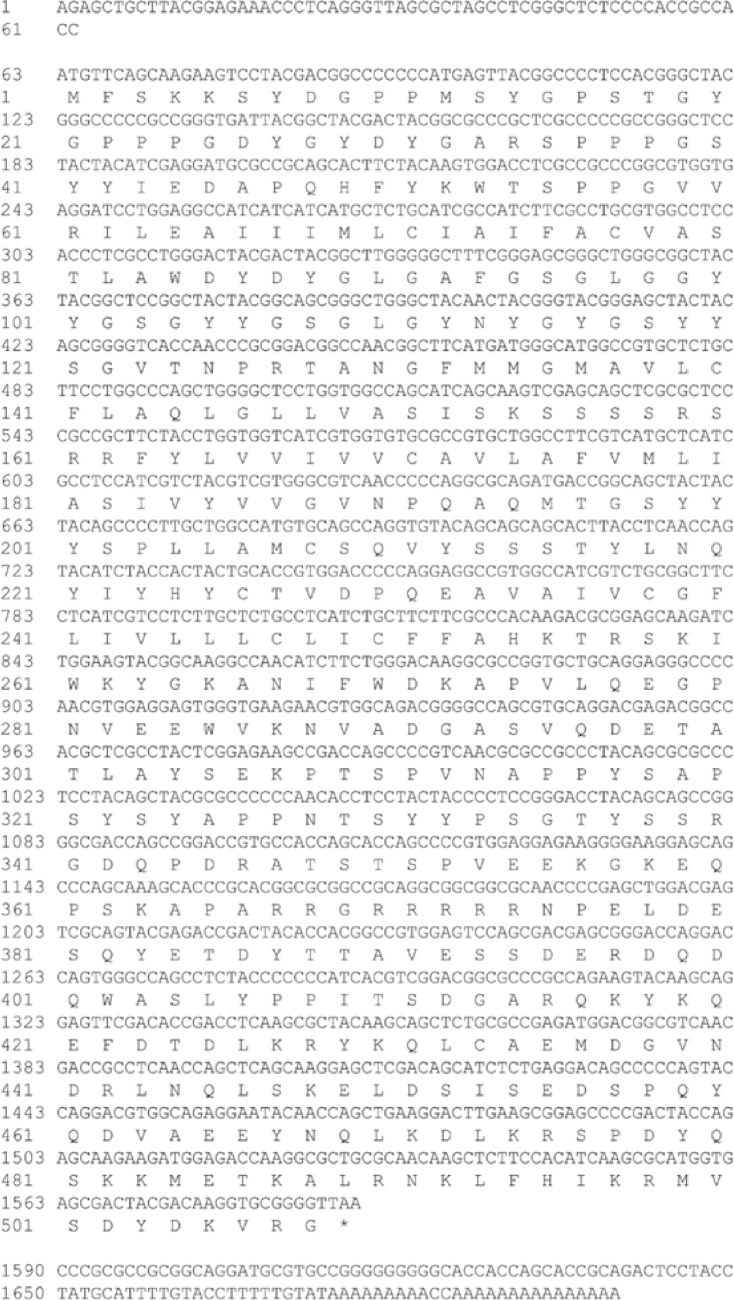
Complete nucleotide sequence encoding occludin and deduced amino acids of the cloned occludin in Muscovy duck (*Cairna moschata*). The asterisk (*) represents the termination codon.

**Figure 2:**
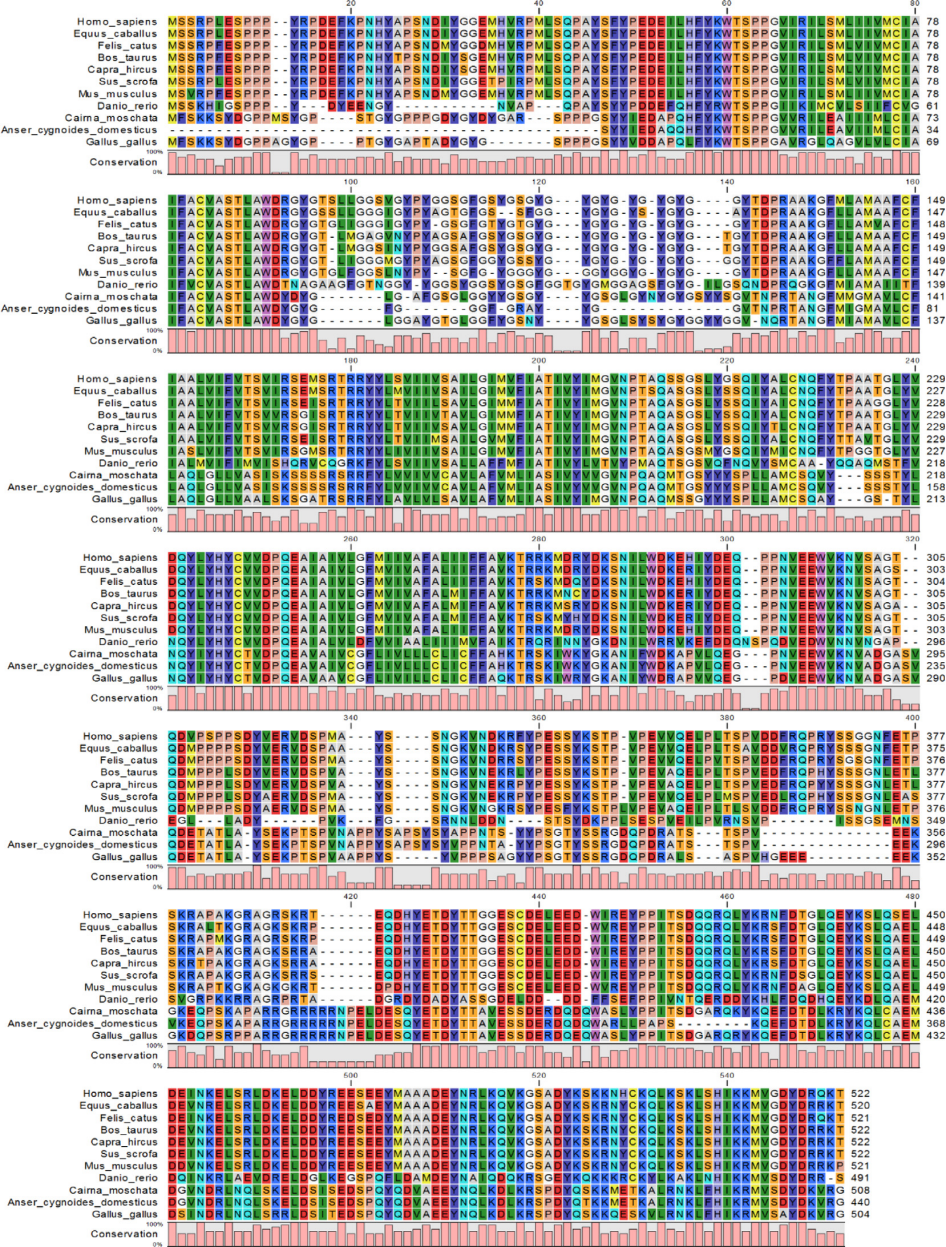
Multiple sequence alignment of occludin gene. The amino acid sequence was used as template to identify homologous vertebrate sequences in PSI-BLAST. The output of Multiple Sequence Alignment was color-coded according to their identity. The phylogenetic tree was constructed by MEGA 5.2 using the neighbor-joining (NJ) algorithm and the reliability of the branching was tested using bootstrap resampling (1,000 pseudoreplicates). The height of the pink bar under the residues represents the conservation of respective residue.

**Figure 3:**
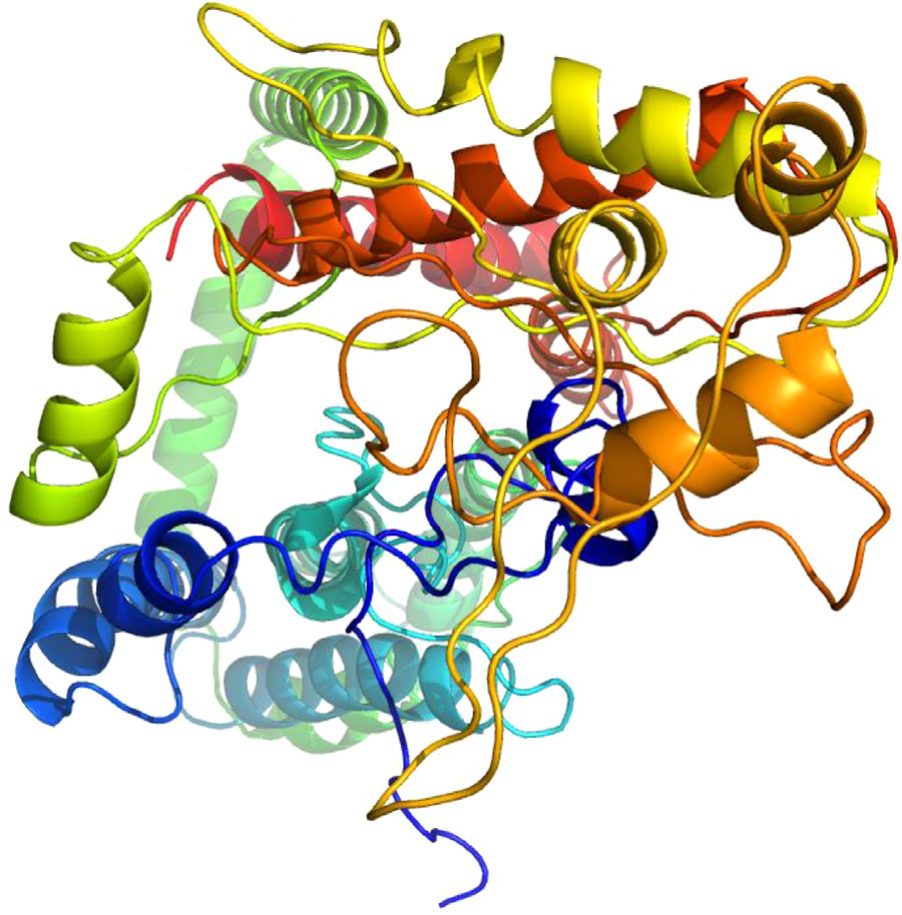
Predicted 3D structure model of Muscovy duck (*Cairna moschata*) occludin. The 3D homology structure modeling of OCLN was predicted by I-TASSER server according to the template of 3G7C and regions of sequence in order from the N (blue) to the C (red) terminus. 3G7C is the crystal structure from Protein Data Bank (PDB) which is Human OCLN gene. The result model (C-score= -0.86) revealed overall folding and secondary structures highly similar to 3G7C. Structural similarity of 2 protein models of the TM-score is 0.61 ± 0.14.

The full cDNA fragment of the occludin gene was obtained using the RACE approach (Figure 1). The cDNA was 1699 bp in length and contained a 1527-bp open reading frame (**ORF**), a 62-bp 5’ untranslated region and a 110-bp 3’ untranslated region. The full length mRNA sequence of occludin has been submitted to the NCBI database with the registration number MT_420729. The BLASTp analysis indicated that Muscovy duck occludin shares high similarity with occludin from different species. The amino acid sequence of Muscovy duck occludin was 89.126% similar with *Anser cygnoides domesticus* and 79.961% with *Gallus gallus*, indicating that the gene identification was correct (Table 2). Furthermore, the homologous sequences from different species were used for multiple sequence alignment with CLC Genomics Workbench 12 (Figure 2). A sequence alignment between the occludin gene of Muscovy duck and that of other species showed that the amino acid sequence of the Muscovy duck occludin protein was highly similar to that of *Anser cygnoides domesticus* and *Gallus gallus*, suggesting that the function of this may be similar to that of the orthologs in these species. Moreover, predicted 3D structure model of Muscovy duck occludin. The predicted 3D structures of the Muscovy duck occludin protein were homologous to the human protein (Figure 3).

### Phylogenetic Analysis

**Figure 4:**
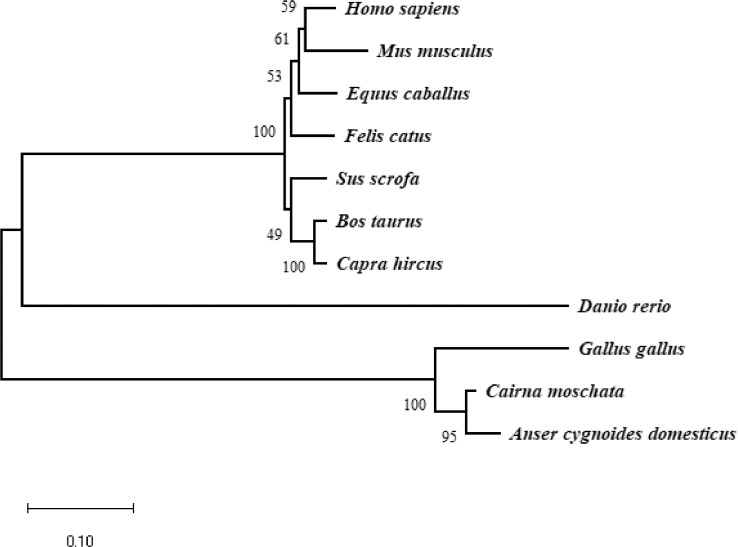
The phylogenetic tree of the Muscovy ducks (*Cairna moschata*) occludin. The protein sequence of Muscovy duck occludin was compared with occludin sequences from other representative species. The phylogenic tree was constructed by MEGA (version 5.2) using the Jones-Thorton-Taylor (JTT) model based upon Neighbor-Joining method with 1,000 bootstrep replicates. The scale bar is 0.10.

To evaluate the evolutionary relationship of the occludin gene in different species, a phylogenetic analysis was performed using the JTT model based upon the neighbor-joining method. The phylogenetic tree of the examined proteins indicated that Muscovy duck, *Anser cygnoides domesticus* and *Gallus gallus* were in the same branch and had high similarity (Figure 4). The results were consistent with the high similarity of the amino acid sequences and indicated that they have a close evolutionary relationship.

### Tissue Expression of the Muscovy Ducks Occludin Gene

**Figure 5:**
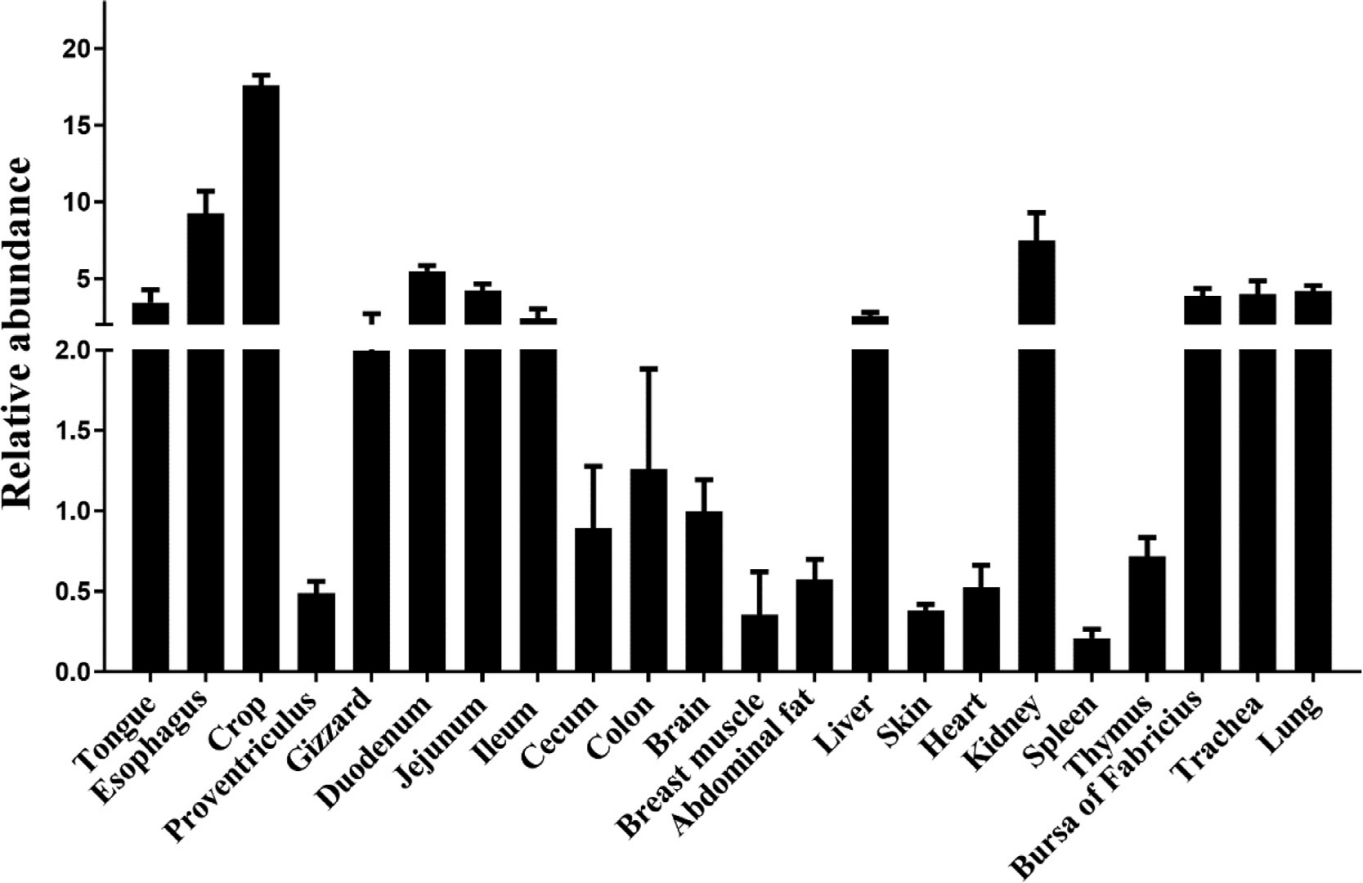
Relative expression levels of occludin in different tissues of Muscovy ducks (*Cairna moschata*). The relative mRNA level of occludin from different tissues of Muscovy duck was quantified by RT-PCR. GAPDH was selected as the internal reference gene. Results were represented as mean ± SEM (n = 8). These results were normalized to the expression level of brain.

To evaluate the gene expression patterns of occludin in various tissues of Muscovy duck, RT-PCR was performed in 22 different tissues (Figure 5). Occludin was expressed in all examined tissues, indicating that it is ubiquitously expressed in various tissues. Specifically, the relative gene expression of occludin was high in the liver, kidney, bursa of Fabricius, trachea, lungs, tongue, esophagus, crop, gizzard, duodenum, jejunum, and ileum. In contrast, the lowest level of occludin expression was detected in the brain, breast muscle, abdominal fat, skin, heart, spleen, thymus, proventriculus, cecum and colon. Furthermore, the occludin gene expression in the intestine showed a trend where the expression in the small intestine was high, while that observed in the large intestine was lower, suggesting the occludin expression in Muscovy ducks was tissue-specific.

### Occludin Gene Expression in Intestinal Tissue of the Muscovy Ducks

**Figure 6:**
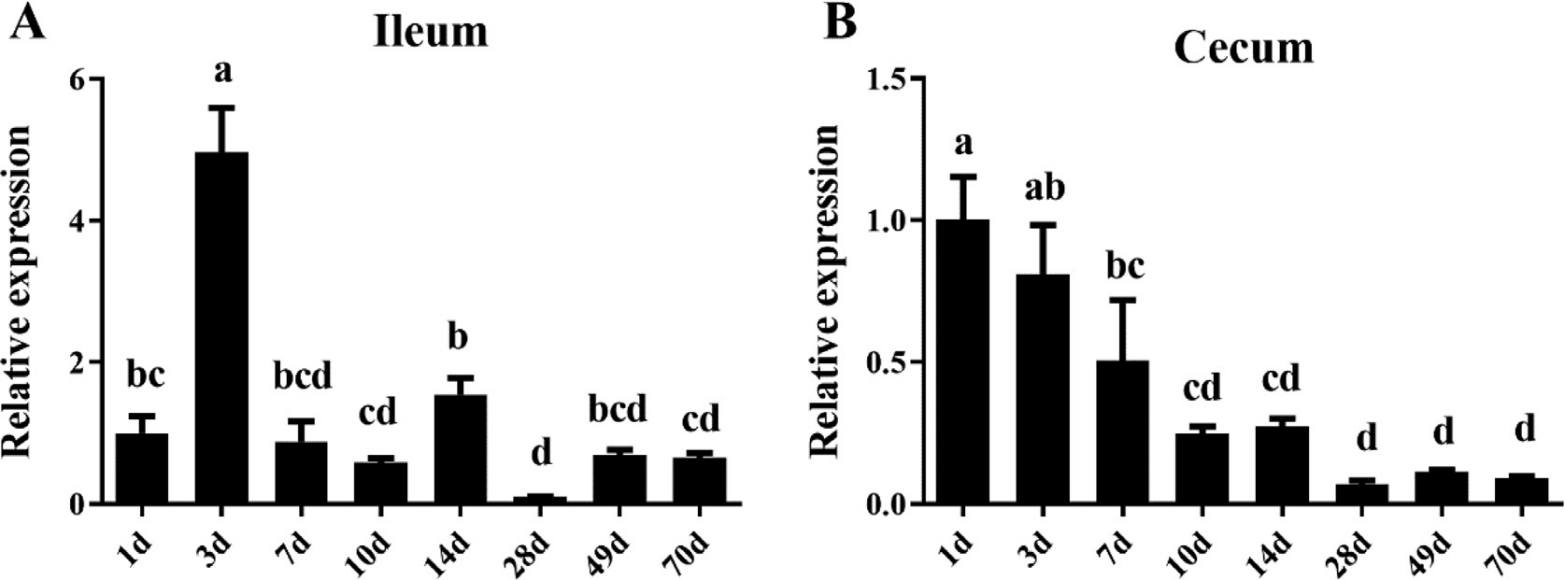
Dynamic expression of occludin in the ileum (A) and cecum (B) of Muscovy ducks (*Cairna moschata*) of different ages. The relative mRNA level of occludin from different tissues of Muscovy ducks was quantified by RT-PCR. GAPDH was selected as the internal reference gene, Results were represented as mean ± SEM (n = 8). The same letter means the difference is not significant, while different letter means the difference is significant. These results were normalized to the expression level of 1th day.

To study the temporal expression of occludin in the GI tract of Muscovy ducks, we collected ileum and cecum segments from Muscovy ducks at 1, 3, 7, 10, 14, 28, 42, and 70 d of age and performed RT-PCR analysis after RNA isolation (Figure 6). A trend of varied occludin gene expression with age was observed. The expression of occludin in the ileum fluctuated and finally stabilized. The expression level of occludin in 3-day-old Muscovy ducks was significantly higher than that observed at the other time points. The results presented in Figure 6 show that the occludin expression level in the 28-day-old ducks was the lowest, but the difference was not significant when compared with that observed in the 49- and 70-day-old Muscovy ducks. In contrast, the expression of occludin in the cecum was the most abundant in the 1-day-old ducks, followed by a decreasing trend before finally becoming stabilized.

### Effects of CB Treatment on the Growth Performance and SCFAs Content in Cecum of Muscovy Ducks

**Figure 7:**
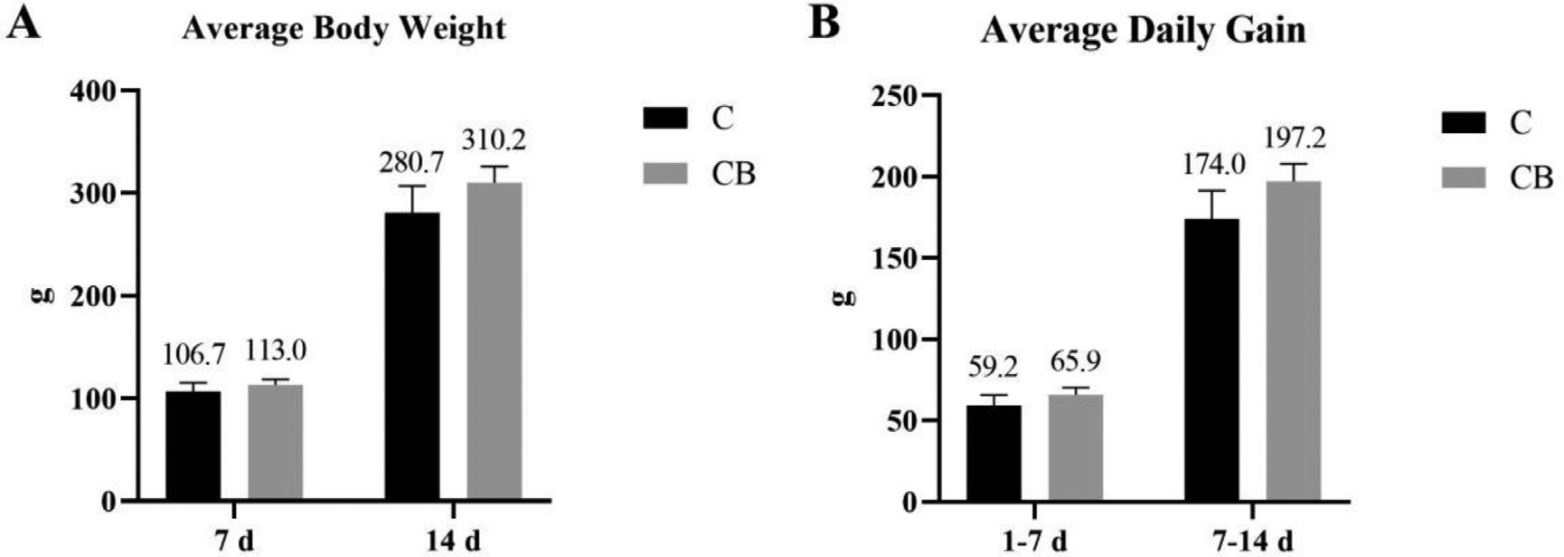
Effects of dietary supplementation with CB on average body weight (A) and average daily gain (B) of Muscovy ducks (*Cairna moschata*). Results were represented as mean ± SEM (n = 8). Abbreviations: C, control group; CB, Clostridium butyricum treated group.

To study whether early intervention with CB would affect the growth performance and of Muscovy ducks, we weighed ducks individually in each group at each time point. As expected, no significant difference between C and CB groups in body weight and average daily feed intake at each time point (*P* > 0.05; Figure 7).

**Figure 8:**
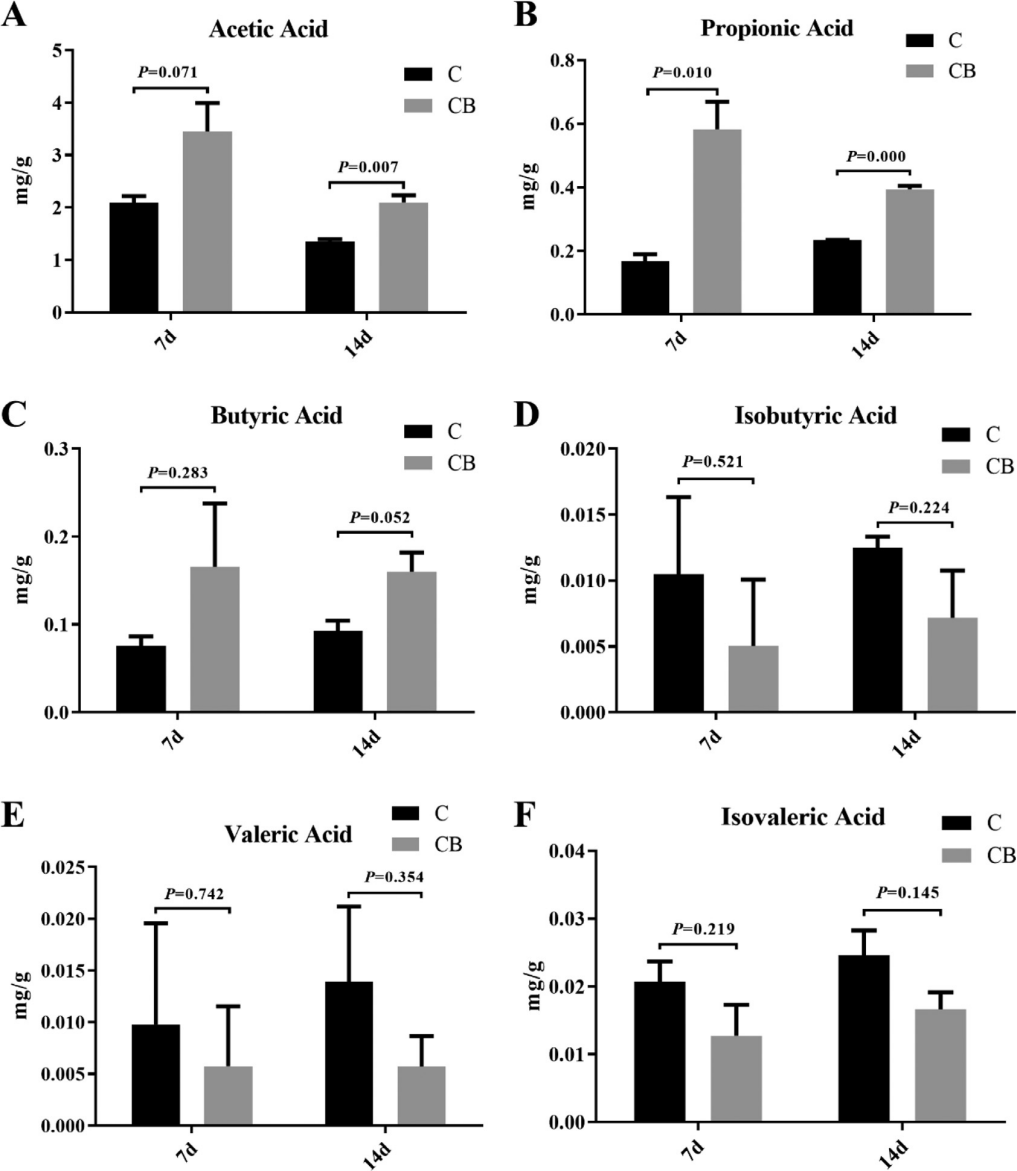
Effects of the oral administration with CB on the concentration of acetic acid (A), propionic acid (B), butyrate acid (C), isobutyrate acid (D), valeric acid (E), and isovaleric acid (F) in the cecal content of Muscovy ducks (*Cairna moschata*). Results were represented as mean ± SEM (n = 8). Abbreviations: C, control group; CB, Clostridium butyricum treated group.

To further investigate whether SCFAs content in cecum of Muscovy ducks would change after early intervention with CB, we examined the concentrations of acetic acid, propionic acid, butyric acid, isobutyric acid, valeric acid, and isovaleric acid in cecal content of each group at d 7 and 14. Generally, acetic acid, propionic acid, and butyric acid were higher in the CB group than in the C group while isobutyric acid, valeric acid, and isovaleric acid gave an opposite trend (Figure 8). In detail, the concentrations of acetic acid and propionic acid in the cecum of CB group were significantly higher than the C group (*P* = 0.007, *P* = 0.000) at d 14 while butyric acid content of the CB group tended to be higher than the C group (*P* = 0.052; Figure 8). Besides, at d 7, the difference in the propionic acid of the cecal content between the C and CB group was significant while the acetic acid concentration of the CB group tended to be significantly higher than the C group at d 7 (*P* = 0.071). There was no significant difference in the butyric acid between the C and CB group (*P* = 0.283). However, there was no significant difference in isobutyric acid, valeric acid, and isovaleric acid content in the cecum between C and CB group (*P* > 0.05) at d 7 and d 14.

### Effect of CB Treatment on the Expression of Occludin in Muscovy Ducks

**Figure 9:**
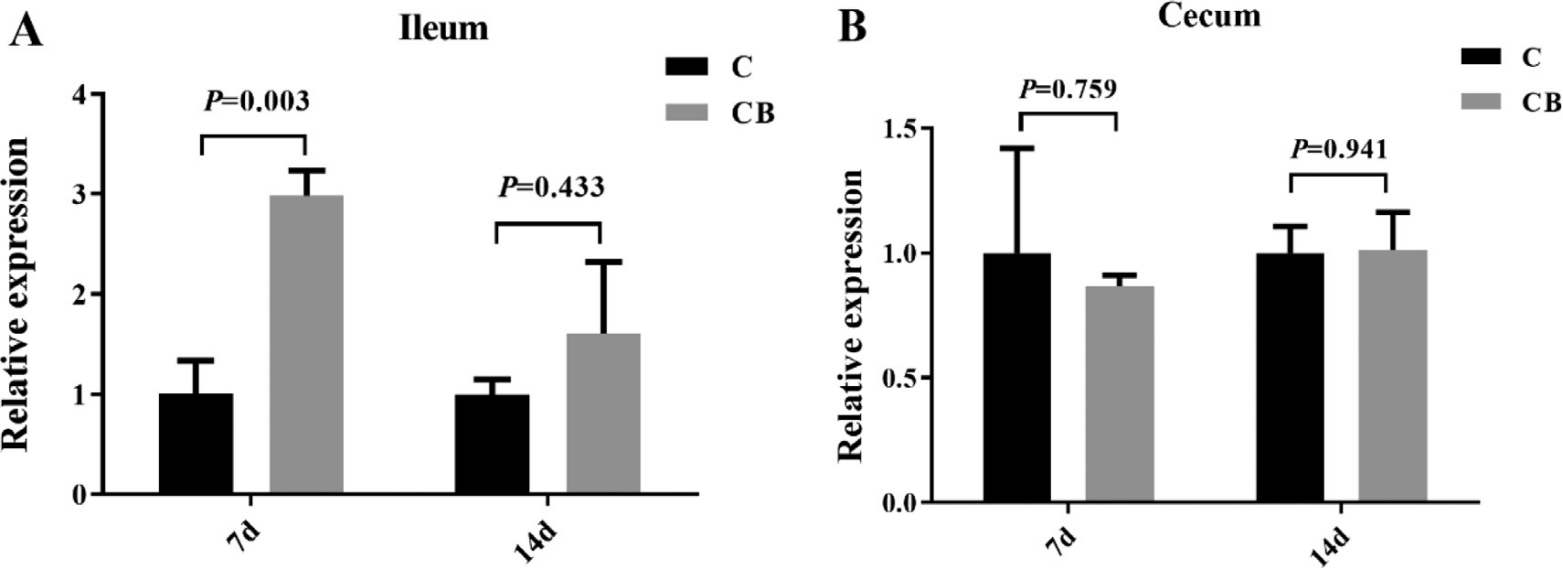
Effects of the oral administration with CB on the expression of Occludin in the ileum (A) and cecum (B) of Muscovy ducks (*Cairna moschata*). GAPDH was selected as the internal reference gene. Results were represented as mean ± SEM (n = 8). *P* < 0.05 was considered as significant. Abbreviations: C, control group; CB, Clostridium butyricum treated group.

We also investigated the effect of the early intervention of CB on the expression of the Muscovy duck occludin gene, the results of which are shown in Figure 9. At the 7th d after the treatment with CB, compared with the control group, the occludin gene expression in the ileum of the CB treatment group ducks was significantly upregulated. In contrast, the change in expression in the cecum was not significant, and there was a downregulated trend. There was no significant difference in the ileum and cecum occludin expression between the 2 groups at the 14th d, but the expression of this gene was upregulated after the addition of CB.

## DISCUSSION

Intestinal diseases are a serious threat to animal health. With the continuous expansion of Muscovy duck breeding, it is of great importance to understand the intestinal barrier function of Muscovy ducks to enhance its resistance to intestinal pathogens. Studies have shown that intestinal occludin is a major component of tight junctions. However, pathogenic *Escherichia coli*, cholera enterotoxin, and rotavirus and *Clostridium difficile* toxins can disrupt the expression of intestinal occludin and cause diarrhea (Fasano et al., 1991; Nusrat et al., 2001; Shifflett et al., 2005; Beau et al., 2007).

Therefore, in the present study, cloning and expression analysis of the occludin gene in Muscovy ducks were reported for the first time. The results showed that the occludin gene was generally expressed in the assayed tissues (Hwang et al., 2013; Ahn et al., 2016). However, the expression was uneven in different tissues. The expression levels were relatively high in the crop and esophagus, possibly because they are the first to contact the feed being at the front end of the digestive tract and as an open organ has close contact with the external environment. As a result, the digestive tracts are easily stimulated by feed components and external factors, resulting in increased occludin gene expression (MacDonald et al., 2003; Brandtzaeg et al., 2004). It is worth noting that occludin was regularly expressed in the intestine, where high expression was observed in the small intestine and was comparatively lower in the large intestine. These results may indicate that occludin is specifically expressed in organs that maintain homeostasis, such as the intestinal tract, and plays a crucial role in the tight junctions of the intestinal tract (Liu et al., 2020). In addition, the dynamic gene expression pattern of intestinal occludin with aging was assessed in this study. The expression of occludin in the ileum showed a fluctuating trend and finally stabilized, and that observed in 3-day-old Muscovy ducks was significantly higher than detected in the other groups. However, the expression of occludin in the cecum was most abundantly expressed in the 1-day-old ducks, followed by a decreasing trend before finally becoming stabilized. Egg-laying animals rely on the yolk to provide nutrition during hatching and continue to absorb the nutrients in the first few days after hatching until they are completely exhausted. The overexpression of the intestinal occludin gene in the early stage may be caused by the oversupply of nutrients from yolk nutrition to promote nutrition (Zhao et al., 2014; Zheng et al., 2014; Zhang et al., 2020). Additionally, the high expression of occludin on d 3 might be due to the early development of ducklings. At the early age of ducklings, they are developing intestinal and other barriers to defense against pathogenic microbe in the environment without the presence of circulating maternal antibodies (Bar-Shira and Friedman, 2006). Especially for the intestine, the intestinal barrier must be developed quickly before the adaptive immunity matures.

Studies have shown that beneficial bacteria can regulate intestinal barrier function (Zhai et al., 2016; Wu et al., 2019). However, CB can secrete antibacterial factors, such as SCFAs and bacteriocins, reducing the pH value of the intestinal environment to prevent pathogenic microorganisms from invading the intestinal epithelial cells, which reduces the inflammatory response and increases the level of occludin expression (Li et al., 2018; Li et al., 2018; Wang et al., 2018). To investigate the dynamic expression pattern of occludin in the intestinal tract of Muscovy ducks with early inoculation of CB, we assessed the expression of occludin in the ileum and cecum in 7- and 14-day-old ducks. The results showed that early inoculation of CB significantly upregulated the expression of occludin in the ileum. This result is consistent with previous research results. The concentration of butyrate is positively correlated with the expression of tight junction proteins, which can maintain and repair the barrier function of the GI tract (Yin et al., 2020; Yu et al., 2020). In contrast, the expression of occludin in the cecum had no significant effect, possibly due to the beneficial effects of other signaling pathways on the intestinal barrier without increasing the expression of tight junction proteins (Suzuki et al., 2011; Zou et al., 2016). However, this possibility requires further investigation.

## CONCLUSIONS

In the present study, we reported the full-length cDNA and bioinformatics analysis of the occludin gene in Muscovy ducks for the first time. The occludin expression was observed to be tissue-specific with the highest expression in the crop of Muscovy ducks. A variation trend of occludin gene expression with age was observed. The temporal expression of occludin in the ileum and cecum indicated that occludin was expressed higher in the early development of Muscovy ducks. Besides, early intervention with CB could significantly increase the occludin expression in the ileum of Muscovy ducks in the first week of Experiment 2 without changes of occludin expression in the cecum. Collectively, these results improve the understanding of occludin dynamic expression in the Muscovy duck intestine and showed the effect of CB on its expression.
